# Cannabis Dampens the Effects of Music in Brain Regions Sensitive to Reward and Emotion

**DOI:** 10.1093/ijnp/pyx082

**Published:** 2017-09-02

**Authors:** Tom P Freeman, Rebecca A Pope, Matthew B Wall, James A Bisby, Maartje Luijten, Chandni Hindocha, Claire Mokrysz, Will Lawn, Abigail Moss, Michael A P Bloomfield, Celia J A Morgan, David J Nutt, H Valerie Curran

**Affiliations:** 1 Clinical Psychopharmacology Unit, University College London, United Kingdom; 2 National Addiction Centre, King’s College London, United Kingdom; 3 Imanova Centre for Imaging Sciences, Imperial College London, Hammersmith Hospital, London, United Kingdom; 4 Neuropsychopharmacology Unit, Division of Brain Sciences, Imperial College London, London, United Kingdom; 5 Institute of Cognitive Neuroscience, University College London, United Kingdom; 6 Behavioural Science Institute, Radboud University, Nijmegen, The Netherlands; 7 Psychiatric Imaging Group, Medical Research Council Clinical Sciences Centre, Hammersmith Hospital, London, United Kingdom; 8 Division of Psychiatry, University College London, United Kingdom; 9 Department of Psychology, University of Exeter, United Kingdom

**Keywords:** cannabis, music, reward, pleasure, emotion

## Abstract

**Background:**

Despite the current shift towards permissive cannabis policies, few studies have investigated the pleasurable effects users seek. Here, we investigate the effects of cannabis on listening to music, a rewarding activity that frequently occurs in the context of recreational cannabis use. We additionally tested how these effects are influenced by cannabidiol, which may offset cannabis-related harms.

**Methods:**

Across 3 sessions, 16 cannabis users inhaled cannabis with cannabidiol, cannabis without cannabidiol, and placebo. We compared their response to music relative to control excerpts of scrambled sound during functional Magnetic Resonance Imaging within regions identified in a meta-analysis of music-evoked reward and emotion. All results were False Discovery Rate corrected (*P<*.05).

**Results:**

Compared with placebo, cannabis without cannabidiol dampened response to music in bilateral auditory cortex (right: *P=*.005, left: *P=*.008), right hippocampus/parahippocampal gyrus (*P=*.025), right amygdala (*P=*.025), and right ventral striatum (*P=*.033). Across all sessions, the effects of music in this ventral striatal region correlated with pleasure ratings (*P=*.002) and increased functional connectivity with auditory cortex (right: *P*< .001, left: *P*< .001), supporting its involvement in music reward. Functional connectivity between right ventral striatum and auditory cortex was increased by cannabidiol (right: *P=*.003, left: *P=*.030), and cannabis with cannabidiol did not differ from placebo on any functional Magnetic Resonance Imaging measures. Both types of cannabis increased ratings of wanting to listen to music (*P<*.002) and enhanced sound perception (*P<*.001).

**Conclusions:**

Cannabis dampens the effects of music in brain regions sensitive to reward and emotion. These effects were offset by a key cannabis constituent, cannabidol.

**Significance Statement**

Significance StatementHere, we report that cannabis administration decreased response to music in several brain regions linked to reward and emotion. These included right ventral striatum, which showed increased functional connectivity with auditory cortex and correlated with pleasure ratings during musical listening, consistent with its critical role in reward processing. These effects were offset when cannabis contained cannabidiol, a key cannabinoid that has been found to reduce some harmful effects of cannabis.

## Introduction

The main psychoactive constituent of cannabis, THC (delta-9-tetrahydrocannabinol), produces subjective effects such as feeling “stoned” and can impair memory and elicit transient psychotic-like symptoms (Curran et al., 2016). Certain types of cannabis also contain cannabidiol (CBD), which can have opposite effects of THC on a range of functional neuroimaging tasks (Bhattacharyya et al., 2010; Batalla et al., 2014). Moreover, CBD has been found to offset harmful effects of THC (e.g., memory impairment and psychotic-like symptoms) without influencing subjective intoxication (Curran et al., 2016; Englund et al., 2017). Cannabis containing high THC and little if any CBD is becoming increasingly prevalent (Hardwick and King 2008; ElSohly et al., 2016) and has been linked to greater mental health and addiction problems compared with less potent varieties of cannabis (Di Forti et al., 2015; Freeman and Winstock 2015).

Despite the changes currently occurring in cannabis legislation worldwide, including legalization of use for medicine and pleasure (Room 2014), few studies have attempted to document the effects that recreational users seek (Curran et al., 2016). The limited evidence of positive effects tends to have arisen incidentally in studies investigating cannabis-related harms. For example, THC has been reported to increase phonological fluency (Curran et al., 2002), a measure of divergent thinking, especially among people with low trait creativity (Schafer et al., 2012).

Cannabis has a strong historical link to music and is associated with several distinct styles, including jazz, reggae, and rock (Booth 2004). Cannabis is reported to enhance appreciation of music (Tart 1970; Green et al., 2003), and its use is consistently high among people who attend music festivals and nightclubs (Lim et al., 2008; Van Havere et al., 2011; Palamar et al., 2015). This association may be partly attributable to shared effects on reward circuitry between drug and nondrug rewards (Berridge and Kringelbach 2015). Music recruits key regions in the reward network, including ventral striatum, mediodorsal thalamus, anterior insula, orbitofrontal cortex, amygdala, and hippocampus (Koelsch 2014).

Many of these reward-related brain regions are characterized by a high density of Cannabinoid Type-1 Receptors (CB1Rs) (Curran et al., 2016). THC is a partial agonist of CB1Rs and may influence response to music by interfering with endogenous CB1R ligands such as anandamide (Thieme et al., 2014), which plays a causal role in consummatory response to reward (Mahler et al., 2007). A human neuroimaging study found that THC (a partial CB1R agonist) dampened the effects of monetary reward feedback across a widespread network, including temporal and orbitofrontal cortices, while leaving reward anticipation intact (van Hell et al., 2012). By contrast, 7-day administration of a CB1R antagonist was found to diminish response to food reward in ventral striatum and orbitofrontal cortex (Horder et al., 2010).

Additionally, THC causes modest, regionally selective increased dopamine release in limbic striatum (Bossong et al., 2015). Such effects might enhance the rewarding experience of music, which can also elicit dopamine release in ventral striatum (Salimpoor et al., 2011) as well as enhancing activation and connectivity between mesolimbic brain regions (Blood and Zatorre 2001; Menon and Levitin 2005; Koelsch et al., 2006; Salimpoor et al., 2011; Trost et al., 2012). Functional connectivity between ventral striatum and auditory cortex during listening also predicts the rewarding experience of music (Salimpoor et al., 2013; Zatorre and Salimpoor 2013; Martínez-Molina et al., 2016).

Here, we conducted the first controlled experimental study on the interactive effects of cannabis and music. Based on previous findings that cannabis and music activate and increase connectivity between common regions in the reward network, whereas a CB1R antagonist dampened neural response to reward (Horder et al., 2010), and observational data linking cannabis use and music, we hypothesized that cannabis would increase haemodynamic response to music in brain regions sensitive to reward and emotion (Koelsch 2014) as well as subjective ratings (wanting to listen to music, pleasure of listening). Given that CBD and THC can have opposing neural effects (Bhattacharyya et al., 2010; Batalla et al., 2014) and CBD can attenuate THC harms (Curran et al., 2016; Englund et al., 2017), we predicted that these effects would be partially offset by CBD.

## Methods

### Design and Participants

A randomized, double-blind, crossover design compared cannabis with CBD (Cann+CBD), cannabis without CBD (Cann-CBD) and matched placebo in 16 cannabis users. Experimental sessions were separated by at least 1 week (>3 times the elimination half-life of THC) to minimize carryover effects (D’Souza et al., 2004; Hindocha et al., 2015). In addition to the music task described here, participants completed additional assessments that are reported elsewhere (Lawn et al., 2016). Inclusion criteria were fluency in English, right-handedness, age between 18 and 70 years, and self-reported current cannabis use (≥4 times in the last year, ≤3 times/wk, ability to smoke a whole joint to oneself). We did not collect data on participants’ typical method of administering cannabis. However, previous data from the UK suggest that the majority (~76%) of cannabis users typically smoke cannabis together with tobacco in joints, and only a small minority (~4%) use a vaporizer as their most common route (Hindocha et al., 2016). Exclusion criteria were self-reported frequent and/or severe adverse reactions to cannabis, current use of illicit drugs other than cannabis more than twice per month, current alcohol use >4 d/wk, significant physical health problems, color blindness, current treatment for a psychiatric disorder, current/history of psychosis, and current/history of psychosis in an immediate family member. This study was approved by the UCL ethics committee, and all participants provided written informed consent.

### Procedure

Following telephone screening, eligible participants completed a baseline session consisting of task training (outside of the MRI scanner), video training for drug administration, drug history (Freeman et al., 2012), and problematic cannabis use on Severity of Dependence Scale (Gossop et al., 1995). The Beck Depression Inventory-II (Beck et al., 1996) and Temporal Experiences of Pleasure (Gard et al., 2006) were also administered. Each experimental session began with a urinary drug screen to verify recent use reported by Timeline Follow-back (Sobell and Sobell 1992). Next, 11-point (0–10) Numerical Rating Scales were administered ~0 minutes before drug inhalation (Pre-Drug), ~5 minutes after first drug administration (Post-Drug), and ~90 minutes after first drug administration (Post-Scan). The Numerical Rating Scales “Want to Listen to Music” and “Enhanced Sound Perception” were administered at all 3 of these time points; “Feel Drug Effect,” “Like Drug Effect,” and “Want More Drug” were administered only after drug administration (Post-Drug and Post-Scan). Heart rate and systolic and diastolic blood pressure were also recorded at the same 3 time points (Pre-Drug, Post-Drug, Post-Scan).

### Drug Administration

Cannabis was obtained from Bedrocan, The Netherlands and used within 6 months of purchase. It was stored on site in foil-sealed pouches at -20ºC and then at ambient temperature prior to drug administration. Each dose was vaporized using a Volcano Medic Vaporizer (Storz and Bickel) at 210ºC in 2 sequentially administered balloons to minimize residual cannabinoids (Lawn et al., 2016). Participants inhaled at their own pace (each inhalation held for 8 seconds, enforced by the experimenter using a stopwatch) until the balloon was empty, which lasted ~5 minutes for both balloons. All participants complied with this administration protocol. Bedrobinol (12% THC, <1% CBD), Bediol (6% THC, 7.5% CBD), and placebo cannabis were used to load doses of 8 mg THC + 10 mg CBD (Cann+CBD), 8 mg THC (Cann-CBD), and placebo (Lawn et al., 2016). Placebo cannabis had a comparable terpene profile to the 2 active forms of cannabis, ensuring it was matched for smell. The same physical quantity of cannabis/placebo (133.4mg) was administered across each of the 3 sessions. This dose of THC has produced effects on brain and behavior in studies with similar vaporizer protocols (Bossong et al., 2009; Hindocha et al., 2015; Mokrysz et al., 2016) and is roughly equivalent to one-quarter of a standard UK joint (Freeman et al., 2014).

### Music Task (Menon and Levitin 2005)

Six 21-second excerpts of standard instrumental classical music were taken from compact disc recordings, adapted from a previous study (Menon and Levitin 2005). Six scrambled versions were created by randomly drawing 250- to 350-millisecond variable-sized sections from each piece and concatenating them with a 30-millisecond linear cross-fade between excerpts (Menon and Levitin 2005). Scrambled excerpts retain the same distribution of pitch and loudness and the same spectral information as normal music. However, they lack temporal structure and are rated as less pleasurable than normal excerpts (Menon and Levitin 2005).

To deliver clear audio during scanning, clips were adapted to improve volume constancy during sections of low volume. Output volume was adapted for each participant in the scanner before the task commenced. Normal/scrambled excerpts were delivered using PsychoPy (Version 1.79.01) through MR compatible sensimetric earphones (http://www.sens.com/products/model-s14/) in a standard blocked fMRI design. The 12 normal/scrambled excerpts were presented in a pseudo-randomized order across the 3 test sessions. Each 21-second excerpt was followed by a 1-second interstimulus interval. Next, participants rated the pleasantness of each excerpt using a 2-finger response pad beneath their right hand (fixed time of 8 seconds). The numerical rating scale was anchored from 0 (not at all pleasant) to 10 (very pleasant). This was followed by 12 seconds of passive fixation (rest). The total task time was 8 minutes 24 seconds, plus a 5-second end-buffer period.

### fMRI Data Acquisition

Imaging data were collected using a Siemens TIM Avanto 1.5T scanner, using a 32-channel receive-only head coil, at the Birkbeck-UCL Centre for Neuroimaging, London. An automated shim procedure was applied to minimize possible magnetic field homogeneities. Functional imaging used a multiband (acceleration factor=4) gradient-echo T2*-weighted echo-planar imaging (EPI) sequence with 40 slices per volume (TR=1000 ms; TE=55 ms; in-plane matrix=64 x 64; 3 mm isotropic voxels; flip angle=75°; bandwidth=1474 Hz/pixel; 509 volumes). The first 8 scans were treated as “dummy” scans and discarded to avoid T1-equilibrium effects. All scanning parameters were selected to optimize the quality of the BOLD signal while maintaining a sufficient number of slices to acquire whole-brain data. To co-register the fMRI data into standard space, we also acquired a MPRAGE structural sequence (TR=2730 ms; TE=3.57 ms; matrix=176 x 256 x 256; 1-mm isotropic voxels; flip angle=7°; bandwidth=190 Hz/pixel; parallel imaging acceleration factor=2), and a B0 field map image (64 axial slices; TR=1170 ms; TE1=10.0 ms; TE2=14.76 ms; in-plane matrix=64 x 64; 3 x 3 x 2 mm voxels; flip angle=90°; bandwidth=260 Hz/pixel) to enable distortion correction of the functional data.

### fMRI Data Analysis

Preprocessing and data analysis were performed using Statistical Parametric Mapping (SPM8; http://www.fil.ion.ucl.ac.uk/spm/software/spm8/). Standard preprocessing procedures consisted of bias correction of EPI images to control for within-volume signal intensity differences, realignment/unwarping to correct for interscan movements, correction for differences in slice acquisition timing, and normalization of the images to an EPI template specific to our sequence and scanner that was aligned to the T1 MNI template. Finally, the normalized functional images were spatially smoothed with an isotropic 8-mm FWHM Gaussian kernel.

At the first level, the normal and scrambled epochs were each modelled as a 21-second boxcar convolved with the canonical hemodynamic response function combined with time and dispersion derivatives to create the contrast music>scrambled, as previously used with this task (Menon and Levitin 2005). The interstimulus interval, rating, and passive fixation (rest) were also modelled. Each subject’s movement parameters were included as confounds. Low-frequency noise was removed with a high-pass filter (cut-off frequency 1/128 Hz). Parameter estimates pertaining to the height of the hemodynamic response function for each regressor of interest were then calculated for each voxel.

At the second level, the contrast music>scrambled was entered into a within-subject ANOVA model with a single factor of drug (Cann+CBD, Cann-CBD, placebo). Within this ANOVA model, we used *t* contrasts to investigate music>scrambled and the reverse contrast (scrambled>music) across all scans. Drug effects on music>scrambled were conducted using *t* contrasts within this ANOVA model. To aid interpretation of significant drug effects (which could reflect changes in response to music, scrambled, or both), separate parameter estimates were extracted from these coordinates for the contrasts music>rest and scrambled>rest using the MarsBaR region of interest toolbox; these were analyzed using repeated-measures ANOVA in SPSS.

Psychophysiological interaction (PPI) analysis was performed to assess task-related functional connectivity (O’Reilly et al., 2012) using seed regions identified by drug effects. We extracted the representative time-course from voxels in the seed region (6-mm radius sphere) using the first eigenvariate calculated from singular value composition. This time course (physiological) was entered into a General Linear Model together with the contrast music>scrambled (psychological) and their interaction (PPI). Motion parameters were included in first-level models as nuisance regressors. The PPI regressor was analyzed using a within-subject ANOVA. We used *t* contrasts to investigate PPI effects across all scans and to compare drug effects.

**Figure 1.**
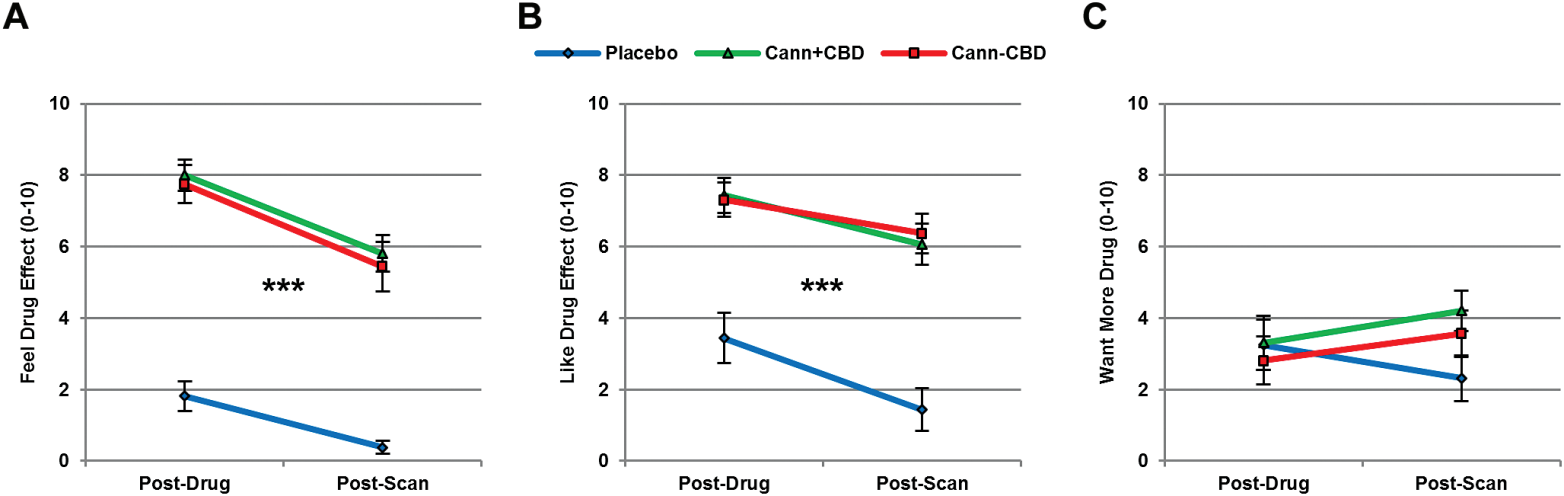
Subjective effects. Both types of cannabis increased ratings for (A) Feel Drug Effect and (B) Like Drug Effect but did not influence (C) Want More Drug. Cann+CBD,cannabis with cannabidiol (CBD); Cann-CBD,cannabis without CBD. ***P<.001.

A False Discovery Rate correction (*P*<.05) was applied to all fMRI analyses. Regions of interest were defined from a previous meta-analysis of music-evoked reward and emotion (see Figure 1 and supplementary Table 1 in Koelsch 2014) using the MarsBaR toolbox. Firstly, each of the structures identified in the meta-analysis was converted into a single sphere. Coordinates were converted to MNI using the Yale BioImage Suite (Lacadie et al., 2008). Sphere radius was estimated from the cluster size reported in the meta-analysis. Where clusters contained multiple structures, size was determined using the cluster mean. Subthreshold clusters were assigned a default size of 200 mm^3^. Each of these spheres was combined into a single mask that was applied to second-level analysis. This mask (41240 mm^3^) included bilateral hippocampal formation, bilateral amygdala, bilateral auditory cortex, right ventral striatum, left caudate nucleus, presupplementary motor area, frontomedian cortex, rostral cingulate zone, pre-genual and middle cingulate cortex, medial and laterial orbitofrontal cortex, right anterior insula, mediodorsal thalamus, and superior parietal lobule.

**Table 1.** Demographic and Drug Use Data

	Mean/ frequency	SD
Age	26.25	7.35
Gender (male/female)	8/8	-
Days of cannabis use per month	8.06	5.48
Years of cannabis use	8.94	7.02
Days since last cannabis use	19.25	45.28
Days to smoke 3.5 g cannabis	25.88	33.73
Severity of dependence scale (cannabis)	1.13	1.26
Alcohol use (yes/no)	16/0	-
Days of alcohol use per month	10.81	4.86
Number of UK alcohol units (8 g) per session	5.93	2.08
Current tobacco use (yes/no)	15/1	-
Days of tobacco use per month	11.30	10.27
Cigarettes per day	3.63	3.62
Current MDMA use <twice a month (yes/no)	6/10	-
Current cocaine use <twice a month (yes/no)	3/13	-
Current ketamine use <twice a month (yes/no)	2/14	-
Beck Depression Inventory-II	3.38	3.12
Temporal experiences of pleasure (anticipatory)	42.06	4.85
Temporal experiences of pleasure (consummatory)	43.50	5.61

### Behavioral Data Analysis

SPSS version 21 was used to analyze all behavioral data and parameter estimates extracted posthoc using MarsBaR. Outliers (>3 times IQR) were winsorized within each session and time point. Histograms were used to investigate normality, and square root or log transformations were applied where appropriate. Trait measures (BDI, Temporal Experiences of Pleasure, SDS, and drug history) were missing for one participant. Missing data from experimental sessions (0.69% of Numerical Rating Scale data, 0.69% of cardiovascular data) were imputed with the mean for that session and time point to retain each participant in the repeated-measures analysis. Repeated-measures ANOVA models were used for all data collected on the 3 experimental sessions, including within-subject factors of drug (Cann+CBD, Cann-CBD, placebo) and time (Pre-Drug, Post-Drug, Post-Scan) or (Post-Drug, Post-Scan) and additional factors where appropriate. Posthoc pairwise tests were Bonferroni-corrected locally within each ANOVA model. Additional repeated-measures ANOVA models were used to aid interpretation of interactions where appropriate. The Greenhouse-Geisser correction was applied where assumptions of sphericity were violated, with degrees of freedom rounded to the nearest integer. To reduce type I and type II error rates, correlations with fMRI data were collapsed across each of the sessions using mixed effects models, with a Bonferroni-adjusted α threshold. These accounted for fixed effects of drug and session order, with a random intercept of participant and maximum likelihood estimation. Equivalent mixed effects models were used to assess possible confounding by cardiovascular measures, cannabis use, and session order.

## Results

### Participants

Seventeen participants completed the study. One participant was excluded due to excessive head movement on one session (exceeding thresholds for both translation [>6 mm] and rotation [>6º]) and was replaced, leaving a final sample of 16. Demographic and drug use data are shown in Table 1. The following number of participants completed each treatment order: Placebo, Cann+CBD, Cann-CBD: n=3; Placebo, Cann-CBD, Cann+CBD: n=2; Cann+CBD, Placebo, Cann-CBD: n=3; Cann+CBD, Cann-CBD, Placebo: n=3; Cann-CBD, Placebo, Cann+CBD: n=2; Cann-CBD, Cann+CBD, Placebo: n=3.

### Behavioral Results

#### Subjective Effects

**Figure 2.**
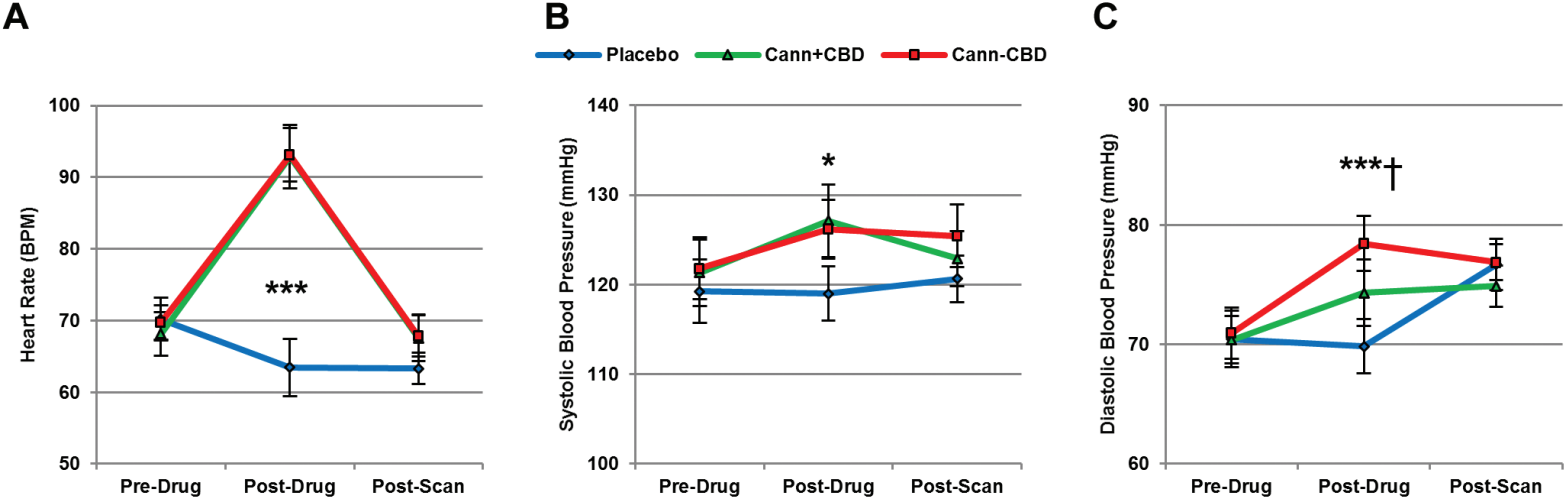
Cardiovascular effects. Both types of cannabis increased (A) heart rate and (B) systolic blood pressure. (C) Diastolic blood pressure increased from Pre- to Post-Drug following cannabis without cannabidiol (CBD), but not following cannabis with CBD; Cann+CBD,cannabis with CBD; Cann-CBD,cannabis without CBD; *P<.05, ***P<.001; ^†^Difference between cannabis types.

Subjective effects are shown in Figure 2 . A main effect of drug (F_1,22_=107.659, *P<*.001, *η*_p_^2^=0.878) emerged for Feel Drug Effect, reflecting increased scores following Cann+CBD (*P<*.001) and Cann-CBD (*P<*.001) compared with placebo, but no differences between Cann+CBD and Cann-CBD (*P=*1.000). There was also a main effect of time, indicating that scores decreased from Post-Drug to Post-Scan (F_1,15_=19.057, *P<*.001, *η*_p_^2^=0.560), but there was no evidence for an interaction between drug and time (F_2, 30_=0.796, *P*=.461, *η*_p_^2^=0.050). Like Drug Effect showed a similar profile of results. There was a main effect of drug (F_2,30_=44.371, *P<*.001, *η*_p_^2^=0.747), reflecting increased scores following Cann+CBD (*P<*.001) and Cann-CBD (*P<*.001) compared with placebo but no difference between Cann+CBD and Cann-CBD (*P=*1.000). There was also a main effect of time, indicating that scores decreased from Post-Drug to Post-Scan (F_1,15_=19.454, *P<*.001, *η*_p_^2^=0.565). Again, there was no evidence for an interaction between drug and time (F_2,30_=0.589, *P=*.561, *η*_p_^2^=0.038). For Want More Drug, there was no evidence for any effects or interactions: drug by time (F_2,30_=2.462, *P=*.102, *η*_p_^2^=0.141), drug (F_2,30_=1.329, *P=*.280, *η*_p_^2^=0.081), or Time (F_1,15_=0.388, *P=*.543, *η*_p_^2^=0.025).

#### Cardiovascular Effects

Cardiovascular effects are shown in Figure 2. For heart rate (BPM), a drug by time interaction emerged (F_2,28_=18.243, *P<*.001, *η*_p_^2^=0.549) as well as main effects of both drug (F_2,30_=13.999, *P<*.001, *η*_p_^2^=0.483) and time (F_2,30_=45.977, *P<*.001, *η*_p_^2^=0.754). Heart rate increased from Pre-Drug to Post-Drug following Cann+CBD (*P<*.001) and Cann-CBD (*P<*.001) but not placebo (*P=*.456). It then decreased from Post-Drug to Post-Scan for both Cann+CBD (*P<*.001) and Cann-CBD (*P<*.001) but did not change on placebo (*P=*1.000). When comparing the 2 types of cannabis alone, there were no differences between the effects of Cann+CBD and Cann-CBD on heart rate across the 3 time points (drug by time interaction: F_2,30_=0.123, *P=*.885, *η*_p_^2^=0.008; main effect of drug: F_1,15_=0.090, *P=*.768, *η*_p_^2^=0.006, main effect of time: F_2,30_=87.391, *P<*.001, *η*_p_^2^=0.854). For systolic blood pressure, a main effect of drug was found (F_2,30_=6.297, *P=*.005, *η*_p_^2^=0.296). This reflected increased blood pressure for both Cann+CBD (*P=*.030) and Cann-CBD (*P=*.006), compared with placebo, but no differences between Cann+CBD and Cann-CBD (*P=*1.000). There was no evidence for an interaction between drug and time (F_4,60_=0.953, *P=*.440, *η*_p_^2^=0.060) or a main effect of time (F_2,30_=2.641, *P=*.088, *η*_p_^2^=0.150). For diastolic blood pressure, an interaction between drug and time was found (F_4,60_=3.217, *P=*.019, *η*_p_^2^=0.177) and a main effect of time (F_2,30_=7.702, *P=*.002, *η*_p_^2^=0.339) but not drug (F_2,30_=2.975, *P=*.066, *η*_p_^2^=0.165). Diastolic blood pressure increased from Pre-Drug to Post-Drug for Cann-CBD (*P<*.001) but not Cann+CBD (*P=*.233) or placebo (*P=*1.000). It then increased from Post-Drug to Post-Scan following placebo (*P=*.030) but not Cann-CBD (*P=*1.000) or Cann+CBD (*P=*1.000).

#### Subjective Music Ratings

**Figure 3.**
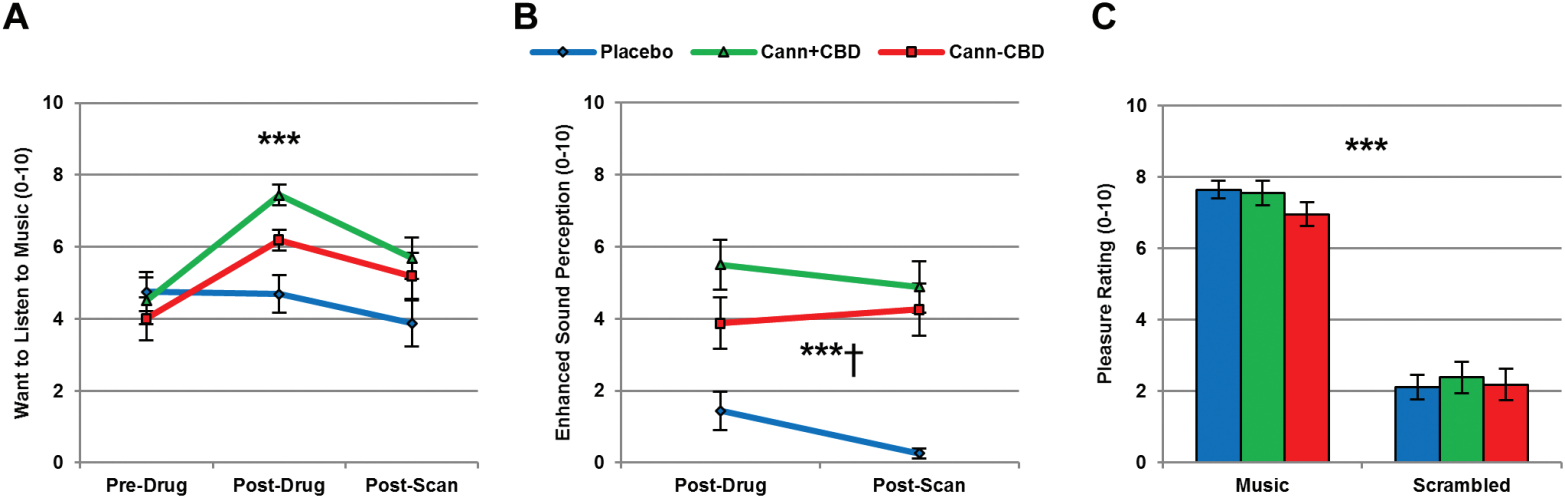
Subjective music ratings. (A) Both types of cannabis increased ratings of Want to Listen to Music. (B) Both types of cannabis increased scores for Enhanced Sound Perception and this increase was greater for cannabis with cannabidiol (CBD). (C) Neither type of cannabis influenced the pleasure of listening to music or scrambled sound clips. Cann+CBD,cannabis with CBD; Cann-CBD,cannabis without CBD. ***P<.001; ^†^Difference between cannabis types.

Subjective music ratings are shown in Figure 3. For Want to Listen to Music, we found a drug by time interaction (F_4,60_=5.256, *P=*.001, *η*_p_^2^=0.259) and main effects of drug (F_2,30_=5.664, *P=*.008, *η*_p_^2^=0.274) and time (F_1,22_=6.300, *P=*.012, *η*_p_^2^=0.296). Scores increased from Pre-Drug to Post-Drug following Cann+CBD (*P<*.001) and Cann-CBD (*P=*.002) but not placebo (*P=*1.000). Scores then decreased from Post-Drug to Post-Scan on Cann+CBD (*P=*.028), but these tests did not reach significance for Cann-CBD (*P=*.553) or placebo (*P=*.199). However, analysis of all three drug conditions suggested that the decrease in Want to Listen to Music from Post-Drug to Post-Scan was equivalent across the 3 sessions (drug by time interaction: F_1,21_=1.130, *P=*.321, *η*_p_^2^=0.070; main effect of drug: F_2,30_=9.158, *P=*.001, *η*_p_^2^=0.379, main effect of time: F_1,15_=7.164, *P=*.017, *η*_p_^2^=0.323). Moreover, when comparing the 2 types of cannabis alone, there were no differences between the effects of Cann+CBD and Cann-CBD on Want to Listen to Music across the 3 time points (drug by time interaction: F_2,30_=0.804, *P=*.457, *η*_p_^2^=0.051; main effect of drug: F_1,15_=3.590, *P=*.078, *η*_p_^2^=0.193, main effect of time: F_2,30_=8.251, *P=*.001, *η*_p_^2^=0.355).

For Enhanced Sound Perception, Pre-Drug scores were removed from analysis due to floor effects on each session. Mean (SD) values were 0.25 (0.45) on placebo, 0.00 (0.00) on Cann+CBD, and 0.00 (0.00) on Cann-CBD. Analysis of variance was therefore restricted to 2 time points (Post-Drug, Post-Scan). A main effect of drug (F_2,30_=44.810, *P<*.001, *η*_p_^2^=0.749) reflected increased scores from placebo following Cann+CBD (*P<*.001) and Cann-CBD (*P<*.001) and higher scores following Cann+CBD compared with Cann-CBD (*P=*.015). There was no evidence for an interaction between drug and time (F_2,30_=2.056, *P=*.146, *η*_p_^2^=0.121) or a main effect of time (F_1,15_=1.248, *P=*.281, *η*_p_^2^=0.077). Finally, we analyzed trial-by-trial pleasure ratings, recorded immediately after listening to classical music and scrambled sound excerpts during MRI scanning. There was a main effect of excerpt (F_1,15_=133.860, *P<*.001, *η*_p_^2^=0.899), indicating that music was rated as more pleasant than scrambled sound. However, there was no evidence for a main effect of drug (F_2,30_=1.205, *P=*.314, *η*_p_^2^=0.074) or a drug by excerpt interaction (F_2,30_=1.221, *P=*.309, *η*_p_^2^=0.075). Next, we calculated a pleasure rating score (music>scrambled) equivalent to our fMRI contrast of interest to provide comparable metrics for brain and behavior. Mean (SD) pleasure rating scores were 5.16 (2.27) for Cann+CBD, 4.78 (2.03) for Cann-CBD, and 5.53 (1.99) for placebo. Analysis of these scores provided no evidence for an effect of drug (F_2,30_=1.221, *P=*.309, *η*_p_^2^=0.075).

### fMRI Results

#### Main Effect of Task

All fMRI analyses were conducted among regions of interest selected from a meta-analysis of previous studies (Koelsch 2014). Across all sessions, listening to music elicited activation in bilateral amygdala, bilateral striatum, left hippocampus, and left cingulate gyrus (see Table 2). For completion, we also examined the reverse contrast (scrambled>music), which revealed activation in bilateral auditory cortex (see Table 2).

**Table 2.** MNI Coordinates for the Contrasts Music>Scrambled (Main Effect of Task, Top Panel) and Scrambled>Music (Main Effect of Task, Middle Panel) across All Sessions. The bottom panel shows brain regions in which participants’ response to music>scrambled was dampened following cannabis without CBD compared with placebo; +: additional peak within cluster. All *P* values are thresholded at *P*<.05 (FDR-corrected for multiple comparisons)

	x	y	Z	mm^3^	Z	*P*
Main effect of task (music>scrambled)						
L Caudate	-12	6	6	540	4.45	.006
L Amygdala	-15	-3	-15	486	4.32	.006
L Hippocampus	-18	-12	-18	+	3.59	.027
R Caudate/thalamus	9	3	6	594	3.91	.014
R Pallidum	15	-3	-6	54	3.33	.031
L Cingulate gyrus	-6	-15	42	27	3.16	.035
R Amygdala	18	-3	-18	54	2.99	.040
Main effect of task (scrambled>music)						
R Planum temporale	60	-12	3	3834	6.61	<.001
R Planum temporale	54	-24	6	+	6.54	<.001
L Planum temporale	-48	-33	9	2511	6.16	<.001
L Heschls gyrus	-42	-24	3	+	5.26	<.001
Drug effect (placebo>cannabis without CBD)						
R Superior temporal gyrus	51	-27	6	2484	4.51	.005
R Planum temporale	60	-12	3	+	3.55	.016
R Planum temporale/heschls gyrus	42	-18	0	+	3.15	.026
L Planum temporale	-42	-33	9	972	4.04	.008
R Hippocampus/parahippocampal gyrus	33	-18	-24	81	3.23	.025
R Amygdala	27	3	-27	27	3.19	.025
R Ventral striatum	15	15	-12	54	2.90	.033

#### Drug Effects

**Figure 4.**
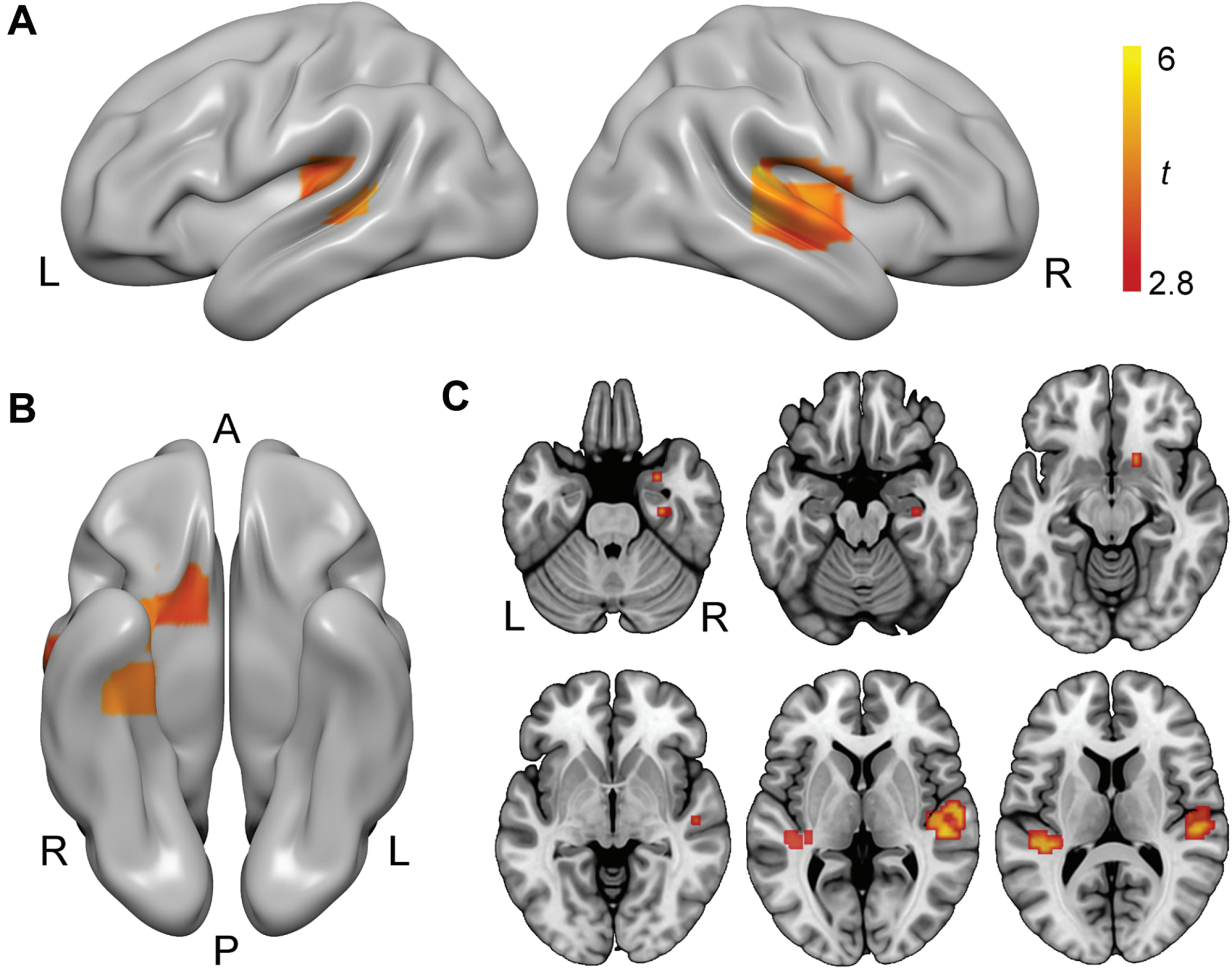
Cannabis without cannabidiol (CBD) dampened brain response to music across several regions sensitive to music-evoked reward and emotion. (A) Bilateral auditory cortex activation clusters visualized on the cortical surface of a standard template (MNI152). (B) A ventral view of the same template showing right-hemisphere amygdala and hippocampal clusters. (C) Axial slice views of the same contrast showing amygdala, hippocampal, ventral striatal (top row), and auditory cortex (bottom row) activation clusters. All activation maps thresholded at *P*<.05 (FDR corrected for multiple comparisons). A,anterior; L, left hemisphere; P,posterior; R, right hemisphere.

Response to music>scrambled was greater on placebo compared with Cann-CBD in bilateral auditory cortex, right hippocampus/parahippocampal gyrus, right ventral striatum, and right amygdala (see Table 2 and Figure 4). There was no evidence for any differences when comparing Cann+CBD with placebo or Cann+CBD with Cann-CBD.

To aid interpretation of these findings (which may have been driven by drug effects on music, scrambled sound, or both), we extracted parameter estimates from each of the clusters identified in this drug effect (Table 2, bottom panel) for separate contrasts of music>rest and scrambled>rest. ANOVA revealed an interaction between drug (placebo, Cann-CBD) and contrast (music>rest, scrambled>rest) (F_1,15_=37.851, *P<*.001, *η*_p_^2^=0.716). This interaction indicated that relative to placebo, Cann-CBD decreased parameter estimates for music>rest (*P=*.009, mean difference -0.195, standard error 0.065). However, there was no evidence for drug effects on scrambled>rest (*P=*.130, mean difference 0.103, standard error 0.064). There were no other findings involving drug (drug by contrast by region interaction: F_4,60_=0.687, *P=*.604, *η*_p_^2^=0.044; drug by region interaction: F_4,60_=0.919, *P=*.459, *η*_p_^2^=0.058; main effect of drug: F_1,15_=0.585, *P=*.456, *η*_p_^2^=0.038). This suggests that Cann-CBD dampened response to music to a similar extent across each of these regions (right auditory cortex, left auditory cortex, right hippocampus/parahippocampal gyrus, right ventral striatum, and right amygdala) while having negligible effects on response to scrambled sound.

#### Brain-Behavior Correlations

**Figure 5.**
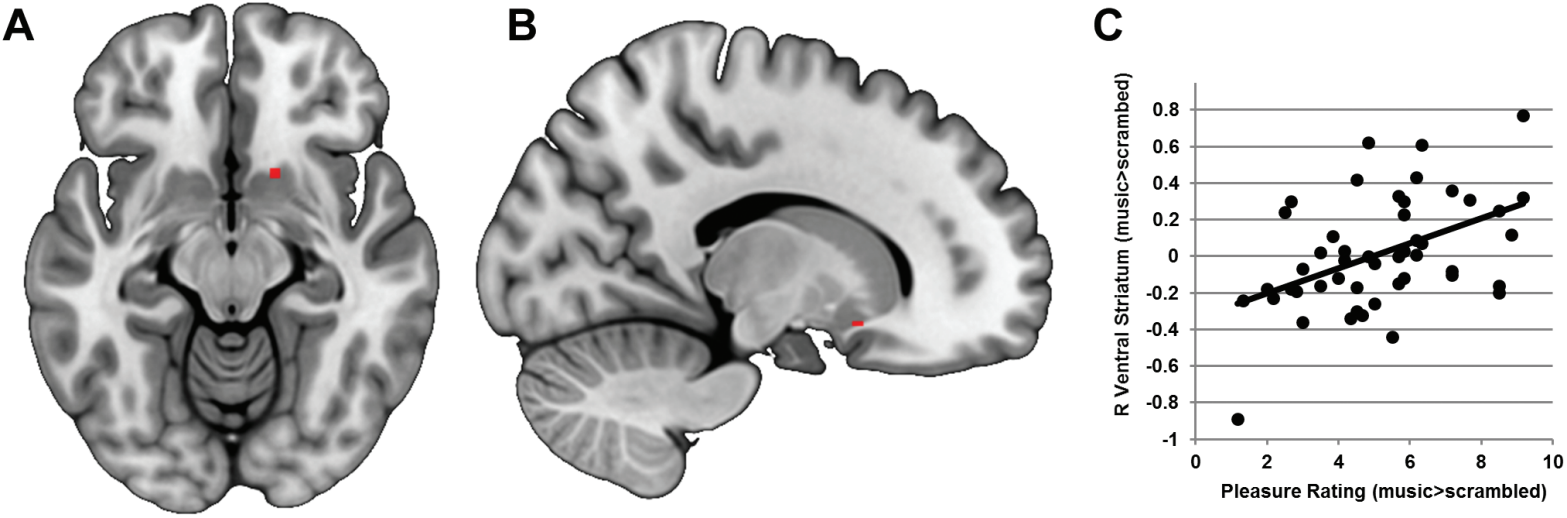
Correlation between brain and behavior. (A) Axial slice of right ventral striatal region of interest, identified from voxelwise analysis. (B) Sagittal slice of the same region. (C) Across all scans, activation in right ventral striatum for the contrast music>scrambled correlated positively with pleasure ratings.

Next, we sought to examine correlations between brain (music>scrambled, extracted from the 5 clusters shown in the bottom panel of Table 2) and behavior (pleasure ratings for music>scrambled, Post-Drug Want to Listen to Music and Enhanced Sound Perception). Data were combined across all sessions to minimize type I and type II error using mixed effects models, resulting in a total of 15 correlations. One correlation reached statistical significance. This showed a positive relationship between pleasure ratings and response to music in right ventral striatum (F_1,34_=11.447, *P=*.002; Figure 5). The same relationship was found using a Pearson correlation analysis across all scans (r=0.463, *P=*.001). This correlation did not contain any outlying values (all data points were <3 times the interquartile range). However, there was still evidence for a correlation after excluding the 2 data points showing the highest and lowest right ventral striatum response to music (mixed effects model: F_1,41_=4.438, *P=*.041; Pearson correlation analysis r=0.318, *P=*.032).

#### Functional Connectivity

**Figure 6.**
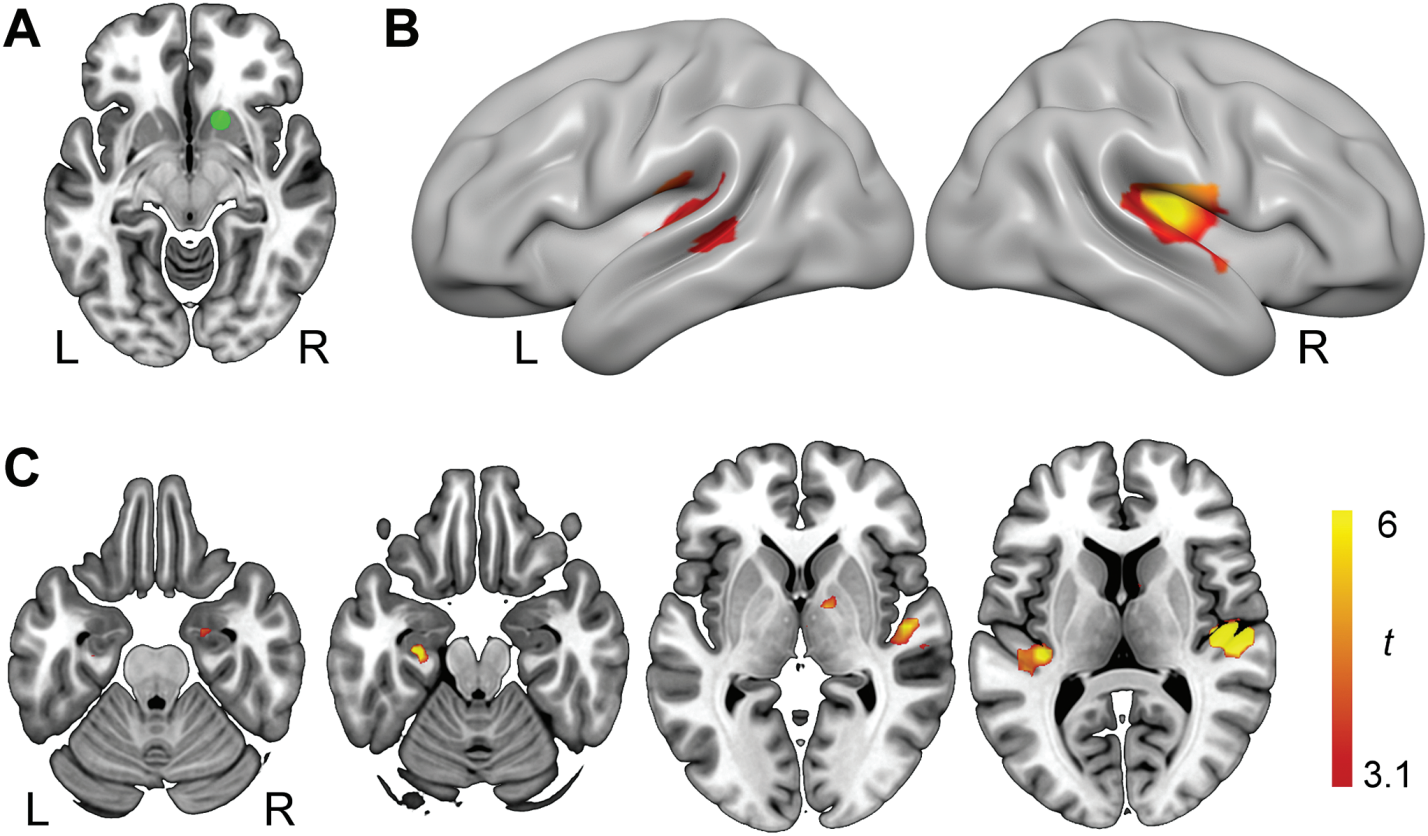
Functional connectivity analysis. (A) Seed region in right ventral striatum. (B) This seed region showed increased task-related functional connectivity with bilateral auditory cortex following cannabis with cannabidiol (CBD) compared with cannabis without CBD (C). Axial slices depicting the same data in bilateral auditory cortex and additional left hippocampal cluster. All activation maps visualized on MNI152 and thresholded at *P*<.05 (FDR corrected for multiple comparisons). L, left hemisphere; R,right hemisphere.

Previous research has shown that the rewarding experience of music is predicted by increased functional connectivity between right ventral striatum and auditory cortex (Salimpoor et al., 2013; Zatorre and Salimpoor 2013; Martínez-Molina et al., 2016). To test this, we conducted PPI analyses. These analyses were conducted posthoc, informed by our findings that Cann-CBD blunted participants’ response to music in right ventral striatum and auditory cortex. Within-subjects ANOVA revealed that across all sessions, the right ventral striatum region (15, 15, -12) identified in our analysis showed a robust increase in functional connectivity with bilateral auditory cortex (and to a lesser extent, right caudate) during music relative to scrambled sound (Table 3). For completion, we conducted the reverse contrast. However, we found no evidence for any regions showing reduced connectivity with this region during music relative scrambled sound. Next, we examined drug effects using *t* contrasts. Compared with Cann-CBD, greater functional connectivity occurred on Cann+CBD between right ventral striatum and bilateral auditory cortex (Table 3; Figure 6). We also conducted a PPI analysis using an auditory cortex seed (right superior temporal gyrus 51, -27, 6). However, this PPI analysis did not identify any regions that showed increases in functional connectivity with the seed region.

**Table 3.** Functional Connectivity Analysis. MNI coordinates showing increased functional connectivity with right ventral striatum for music>scrambled across all sessions (main effect, top panel). Functional connectivity between right ventral striatum and auditory cortex increased on cannabis with CBD compared with cannabis without CBD (drug effect, bottom panel); +Additional peak within cluster. All *P* values are thresholded at *P*<.05 (False Discovery Rate-corrected for multiple comparisons)

	X	y	z	mm^3^	Z	*P*
Main effect						
R Planum temporale	60	-12	6	5319	6.43	<.001
R Heschls gyrus/planum polare	48	-12	0	+	6.42	<.001
R Planum temporale	48	-27	9	+	6.26	<.001
L Heschls gyrus	-42	-24	12	3429	6.31	<.001
L Planum temporale	-42	-30	6	+	6.27	<.001
L Planum temporale	-33	-33	15	+	5.48	<.001
R Caudate	9	15	9	54	2.42	.037
Drug effect (cannabis with CBD>cannabis without CBD)						
R Heschls gyrus	42	-18	9	1620	4.63	.003
L Hippocampus	-30	-18	-21	81	3.64	.009
L Heschls gyrus	-36	-27	9	54	3.08	.030
L Heschls gyrus	-45	-24	15	27	3.05	.031

### Possible Confounding

#### Cardiovascular Drug Effects

We conducted correlations between all Post-Drug cardiovascular measures (heart rate, systolic and diastolic blood pressure) and the 9 clusters showing evidence of drug effects (see Tables 2 and 3) across all sessions (total 27 correlations). We found no evidence for any association between cardiovascular and fMRI data (all *P*>.05).

#### Cannabis Use

We also explored correlations between levels of cannabis of use and our main findings. These were conducted between (1) years of cannabis use, (2) days of cannabis use per month and the 9 clusters showing evidence of drug effects (Tables 2 and 3), Want to Listen to Music (Post-Drug), Enhanced Sound Perception (Post-Drug), and pleasure rating scores. Of the 24 correlations, we found no evidence for any associations (all *P*>.05) apart from a trend negative correlation between years of cannabis use and functional connectivity between right ventral striatum and left hippocampus (F_1,48_=4.984, *P=*.030). However, this did not reach significance at a Bonferroni-corrected threshold (α=0.0021). Moreover, the effect of drug remained significant in this model (F_1,48_=7.455, *P=*.002).

#### Order Effects

Because the same music and scrambled sound excerpts were presented across each of the 3 sessions, we investigated possible order effects. For all fMRI results showing drug effects, the effect of drug remained significant, and there was no evidence for an effect of session order (all *P*>.05). There was no evidence for effects of drug or session order for pleasure rating scores (all *P*>.05). Analysis of Want to Listen to Music (Post-Drug) and Enhanced Sound Perception (Post-Drug) scores showed effects of drug (both *P*<.001) but not session (both *P*>.05).

## Discussion

To our knowledge, this is the first controlled experiment investigating the interactive effects of cannabis and music. Cannabis dampened response to music in several regions implicated in music-evoked reward and emotion (Koelsch 2014): bilateral auditory cortex, right amygdala, right hippocampus/parahippocampal gyrus, and right ventral striatum. Across all scans we found a positive correlation between response to music in this ventral striatal region and the pleasure of listening to the same sound clips, consistent with several studies implicating the ventral striatum in musical pleasure (Blood and Zatorre 2001; Koelsch et al., 2006; Salimpoor et al., 2011; Trost et al., 2012). The same ventral striatal region showed increased task-related functional connectivity with bilateral auditory cortex, an effect that has previously been shown to predict musical reward value (Salimpoor et al., 2013; Zatorre and Salimpoor 2013; Martínez-Molina et al., 2016).

These findings were contrary to our prediction that cannabis would increase the rewarding effects of music, which can activate and increase connectivity within mesolimbic brain regions (Blood and Zatorre 2001; Menon and Levitin 2005; Koelsch et al., 2006; Salimpoor et al., 2011; Trost et al., 2012) and, in common with THC, may increase striatal dopamine release (Salimpoor et al., 2011; Bossong et al., 2015). Moreover, observational data suggests that cannabis is frequently used in the context of music and may enhance its effects (Tart 1970; Green et al., 2003; Lim et al., 2008; Van Havere et al., 2011; Palamar et al., 2015).

One possible explanation for our findings is that THC interfered with the endocannabinoid system, which plays a critical role in reward processing (Parsons and Hurd 2015). For example, acute THC may deplete the CB1R ligand anandamide (Thieme et al., 2014), which increases consummatory response to reward in the nucleus accumbens shell (Mahler et al., 2007). Disruption of the endocannabinoid system could explain why neural response to reward was previously dampened by 7-day administration of a CB1R antagonist (Horder et al., 2010) as well as a single dose of the partial CB1R agonist THC (van Hell et al., 2012). It should also be noted that our findings of dampened response to music occurred in the context of increased wanting to listen to music. These findings are broadly consistent with previous findings that THC may have dissociable effects on anticipatory (“wanting”) and consummatory (“liking”) components of reward (van Hell et al., 2012; Jansma et al., 2013), although our task lacked a neural index of reward anticipation.

Cannabis with CBD did not differ from placebo on any fMRI measures. Furthermore, it resulted in greater task-related functional connectivity between ventral striatum and auditory cortex compared with cannabis without CBD. These findings suggest that CBD was able to offset some effects of THC, consistent with previous research (Curran et al., 2016; Englund et al., 2017) and evidence that THC and CBD can have opposite neural effects (Bhattacharyya et al., 2010; Batalla et al., 2014). For example, activation in right superior temporal gyrus (a region identified in our study) during word listening relative to rest was previously found to be decreased by THC but increased by CBD (Winton-Brown et al., 2011). Moreover, CBD may increase concentrations of anandamide (Bisogno et al., 2001; Leweke et al., 2012). We found some evidence that CBD interacted with THC on additional measures. Taken together, CBD appeared to partially offset some negative effects of THC (increase in diastolic blood pressure, decreased response to music) while preserving or potentiating desirable ones (enhanced sound perception, functional connectivity with ventral striatum during musical listening).

In terms of clinical implications, the effects of acute cannabis administration here are similar to previous findings in people with depression, who also show a blunted response to music in ventral striatum as well as medial orbitofrontal cortex (Osuch et al., 2009). In this respect, acute cannabis administration may transiently mimic the diminished response to reward characteristic of some mental health disorders. The impact of chronic cannabis administration remains unclear. However, a 4-year prospective study found that increased cannabis use was associated with subsequent reductions in ventral striatal response to reward anticipation (Martz et al., 2016). It therefore is possible that effects of cannabis on reward processing may contribute to an increased risk of developing depression (Zhang et al., 2013; Lev-Ran et al., 2014) as well as other disorders characterized by reward dysfunction such as addiction and psychosis (Radua et al., 2015; Luijten et al., 2017). Moreover, our findings support the potential utility of CBD in reducing cannabis harms while maintaining the positive effects users seek (Englund et al., 2017).

Strengths of this study include its controlled experimental design, comparison of cannabis with and without CBD (but matched for THC), a music task previously validated using fMRI (Menon and Levitin 2005), and regions of interest informed by meta-analysis (Koelsch, 2014). Our sample size was equivalent or larger than previous studies with comparable designs (van Hell et al., 2012; Jansma et al., 2013) and neuroimaging music studies of music (mean n=14.5 across 22 studies (Koelsch 2014)). We used a fixed set of classical music excerpts, commensurate with previous use of this task (Menon and Levitin, 2005) and many other studies (Koelsch, 2014). Advantages of this approach include the absence of lyrics (which would influence neural response due to speech) and ease of comparison with existing data. Although participants rated classical sound clips as highly pleasant (~7.5 of 10), results may have differed if preferred music was preselected by participants (Osuch et al., 2009). Drug order was not completely balanced in this study, and the same sound clips were presented on each of the 3 sessions. However, we found no evidence that session order influenced our results. We screened for personal/family history of psychosis and current treatment for a psychiatric disorder, but not for lifetime history of other mental health problems. Additionally, our sample were cannabis users, which may have prolonged cannabinoid clearance between sessions. However, we found minimal evidence for associations between cannabis use and fMRI findings, and their response to cannabinoids may be more representative of typical use than healthy volunteers who never use cannabis.

In conclusion, cannabis dampened the effects of music in bilateral auditory cortex, right hippocampus/parahippocampal gyrus, right amygdala, and right ventral striatum. During musical listening, this ventral striatal region correlated with pleasure ratings and showed increased functional connectivity with auditory cortex. By contrast, cannabis containing cannabidiol did not influence the effects of music in brain regions sensitive to reward and emotion.

## Funding

T.P.F. was funded by the UK Medical Research Council and a Senior Academic Fellowship from the Society for the Study of Addiction. This study was funded by Drug Science/Channel 4 television.

## Statement of Interest

H.V.C. is a member of UK MRC boards and Drug Science. D.J.N. is an advisor to the British National Formulary, MRC, GMC, Department of Health; President of European Brain Council; Past President of British Neuroscience Association and European College of Neuropsychopharmacology, Chair of Drug Science (UK); Member of International Centre for Science in Drug Policy; advisor to Swedish government on drug, alcohol, and tobacco research; editor of the Journal of Psychopharmacology; member of advisory boards of Lundbeck, MSD, Nalpharm, Orexigen, Shire, MSD; has received speaking honoraria (in addition to above) from BMS/Otsuka, GSK, Lilly, Janssen, Servier, AZ, and Pfizer; is a member of the Lundbeck International Neuroscience Foundation; has received grants or clinical trial payments from P1vital, MRC, NHS, Lundbeck, RB; has share options in P1vital; has been an expert witness in a number of legal cases relating to psychotropic drugs; and has edited/written 27 books, some purchased by pharma companies. C.J.A.M. has consulted for Janssen and GlaxoSmithKline and received compensation. M.B.W. is employed by Imanova Ltd., a private company that performs contract research work for the pharmaceutical industry. The other authors declare no potential conflicts of interest.
